# Comments on "An insertion unique to SARS-CoV-2 exhibits super antigenic character strengthened by recent mutations" by Cheng MH et al.2020

**DOI:** 10.6026/97320630016474

**Published:** 2020-06-30

**Authors:** Francesco Chiappelli

**Affiliations:** 1 UCLA Center for the Health Sciences; CSUN, Department of the Health Sciences, LA, California, USA

## Description:

This pre-print research paper reports [5] an informative experimental study related to the causative factors of the Multisystem Inflammatory Syndrome in Children (MIS-C) associated with Coronavirus Disease 2019 (CoViD-19) in children with recent SARS-CoV-2 infection. MIS-C, like CoViD-19 in adults, presents with life- threatening symptoms of hypotension, multi-organ collapse, and elevated inflammatory markers. The authors noted that the escalation of the cytotoxic adaptive immune response triggered upon super-antigen (S-Ag) presented as non-self by MHC Class-II to T cell receptors (TcRs) resembles the toxic shock syndrome. They therefore tested the hypothesis that the spike protein, S, of SARS-CoV-2 is endowed with S-Ag activity.

The findings of structure-based computational modeling showed that S presents a high-affinity motif for, and interacts closely with both the complementarity-determining regions of the variable domains for both the α- and β-chains of TcR. Further examination determined that the S binding epitope harbors a sequence motif unique to SARS-CoV-2, and not expressed in the preceding SARS-CoV, MERS or any member of the Corona virus family tested. The S motif is similar in both sequence and structure to bacterial S-Ag, and has a selected- residues motif within its interfacial region that mimics the intracellular adhesion molecule (ICAM). ICAM's are molecules of the immunoglobulin super-family that play an important role in inflammation, cell-mediated immune responses in general, and in intracellular signaling events [1]. The data further indicated that TcR-SARS- CoV-2 interaction appears to vary among mutated strains [2], and that it is stronger with the European mutation (D839Y/N/E) compared to the original Asian strain.

Moreover, in silico modeling revealed that the S-Ag motif encoded by SARS-CoV-2 is located near its S1/S2 cleavage site, a region that is remarkably similar in structure to the staphylococcal entero-toxins B S-Ag motif. That specific S-Ag interacts with both TcR and CD28, which provide co-stimulatory signals required for T cell activation and survival. T cell Receptor co-stimulation through CD28 yields a potent signal for the production of various interleukins, including IL-6, IL-2 and other cytokines [3]. The data in this paper therefore suggest that the SARS-CoV-2 S-Ag motif may stimulate massive production of pro-inflammatory cytokines (IL-2, IL-6, IFNg,) from T cells and others (IL-1b, TNFa) from activated antigen-presenting cells, resulting in the 'cytokine storm'. The 'storm' is the major cause of multi-organ tissue damage, collapse and failure in MIS-C, in CoViD-19, as in the toxic shock syndrome [4]. Taken together, the authors propose important implications of their findings of the interaction of the spike, S, protein of SARS-CoV-2 with human TcR α- and β-chains. They propose that these interactions, found to trigger the cytotoxic adaptive immune response as a S-Ag, should become the focus for the development of therapeutic approaches for CoViD-19 in adults and children.

In brief, this is an experimental study with major clinical relevance. The reported findings yield a new understanding of the immuno-pathology leading to severe manifestations of CoViD-19, in adults and children, which is of critical importance for effective management and treatment of the disease, as well as for preventive measures against future waves of SARS- CoV-2 infection. It appears possible and even probable that the promising findings proffered in this study may point to novel immuno-modulatory therapeutic options for patients with CoViD-19 or MIS-C.
